# Unidirectional Fabric Drape Testing Method

**DOI:** 10.1371/journal.pone.0143648

**Published:** 2015-11-24

**Authors:** Zaihuan Mei, Wei Shen, Yan Wang, Jingzhi Yang, Ting Zhou, Hua Zhou

**Affiliations:** Key Laboratory of Advanced Textile Materials and Manufacturing Technology, Ministry of Education, Zhejiang Sci-Tech University, Hangzhou, China; Glasgow University, UNITED KINGDOM

## Abstract

In most cases, fabrics such as curtains, skirts, suit pants and so on are draped under their own gravity parallel to fabric plane while the gravity is perpendicular to fabric plane in traditional drape testing method. As a result, it does not conform to actual situation and the test data is not convincing enough. To overcome this problem, this paper presents a novel method which simulates the real mechanical conditions and ensures the gravity is parallel to the fabric plane. This method applied a low-cost Kinect Sensor device to capture the 3-dimensional (3D) drape profile, thus we obtained the drape degree parameters and aesthetic parameters by 3D reconstruction and image processing and analysis techniques. The experiment was conducted on our self-devised drape-testing instrument by choosing different kinds of weave structure fabrics as our testing samples and the results were compared with those of traditional method and subjective evaluation. Through regression and correlation analysis we found that this novel testing method was significantly correlated with the traditional and subjective evaluation method. We achieved a new, non-contact 3D measurement method for drape testing, namely unidirectional fabric drape testing method. This method is more suitable for evaluating drape behavior because it is more in line with actual mechanical conditions of draped fabrics and has a well consistency with the requirements of visual and aesthetic style of fabrics.

## Introduction

As an important aesthetic indicator of textiles, drape refers to the 3D deformation of fabrics arising from their own weight [1–2] and affects garment appearance quality profoundly [3–4]. Measuring and predicting the behavior of draped fabrics has been one of the most significant things in the field of fabric computer simulation which helps to clothing design and production [5–8].

Normally, drape is subjectively evaluated by textile and apparel workers in the design and manufacturing industry [9], but the result of this method varies greatly with different individuals and lacks of reproducibility. To find an efficient, accurate and reliable method to reflect the drape property, lots of effort and improvements have been made to interpret drape quantitatively. The progress of evaluating fabric drape was first begun by Peirce in 1930 [10]. He developed an instrument and defined two parameters, bending length and flexural rigidity, to quantize draping quality of a fabric instead of judging handle or feel. This is an indirect method to reflect drape. Although this method is supposed to be feasible to measure drape, using two-dimensional distortions measurement and only two parameters to describe 3D complex fabrics is not convincing enough. A modified method was later proposed by Chu *et al* in 1950 [11–12]. He designed the standard Fabric Research Liberating (F.R.L) drape meter which was capable of distorting the fabric in all three dimensions and proposed the drape coefficient for the first time to analyze drape. The F.R.L drape meter is an optical instrument that traces the draped pattern of the circular sample sandwiched between two circular plates on a thin piece of paper, and then the draped pattern is cut and weighed to obtain the drape coefficient. The principle of F.R.L drape meter becomes the basis of most of further developed devices, however it is very time consuming and needs skills [13]. The most commonly applied testing device for fabric drape is the Cusick Drape Meter [14] which is also designed based on the principle of F.R.L drape meter. The Cusick drape meter obtained more accurate drape coefficient with less tedious and costly procedures, but on the other hand, it could not completely describe drape behavior with two-dimensional fabric projection image and only one parameter.

With the development of photography techniques and computer, researchers began investigating the use of image processing technology [13, 15–17] in studying fabric drape on the basis of Cusick drape meter. This method involved a camera and a beam of parallel light to capture the projection of draped fabric and a set of software to process the projection image. By means of image analysis the detailed data of drape property such as drape shape parameters and statistical information including drape wave amplitude, wave length and number of nodes were developed and computed from drape profile image [9]. Image analysis method takes less time, independent of operator, and has a better repeatability with more parameters and makes it possible to investigate time dependence of drape coefficient [18].

In view of previous work, we can notice that both the cut and weigh devices (F.R.L drape meter and Cusick drape meter) and image analysis equipments are based on umbrella projection method, namely traditional method, which makes the sample draped on a circular plate and captures its contour to evaluate drape. This method predicts and evaluates drape using a two-dimensional projection image, but fabric drape is a 3D deformation property [19] and different samples may have the same projection contour. More important, it also ignores a key aspect that the fabric is subjected to gravity perpendicular to its plane, which is contrary to most actual cases that the fabrics such as curtains, skirt, suit pants etc. are normally draped under their gravity parallel to the plane. So the traditional method is not appropriate and accurate enough, and a novel method is necessary.

Some researchers made attempt to test fabric drape with the method of 3D reconstruction, but they still use umbrella model to capture 3D contour of fabric [20–21]. Although the commercial 3D scanners can capture the 3D fabric model, they are normally expensive and not suitable for drape testing.

This paper proposed a new, low-cost and more reasonable method, the unidirectional drape testing method, which imitates the actual mechanical conditions of draped fabric to analyze drape performance. In this study, we designed a corresponding drape meter and established unidirectional fabric drape evaluation system from the real 3D draped contour. To verify the feasibility of our new method, we compared the accuracy of measurements from unidirectional method with those of the conventional method. In addition, a comparison was made with subjective evaluation to further prove the feasibility of this method.

## Experimental Details

### Equipment

**Fig 1:**
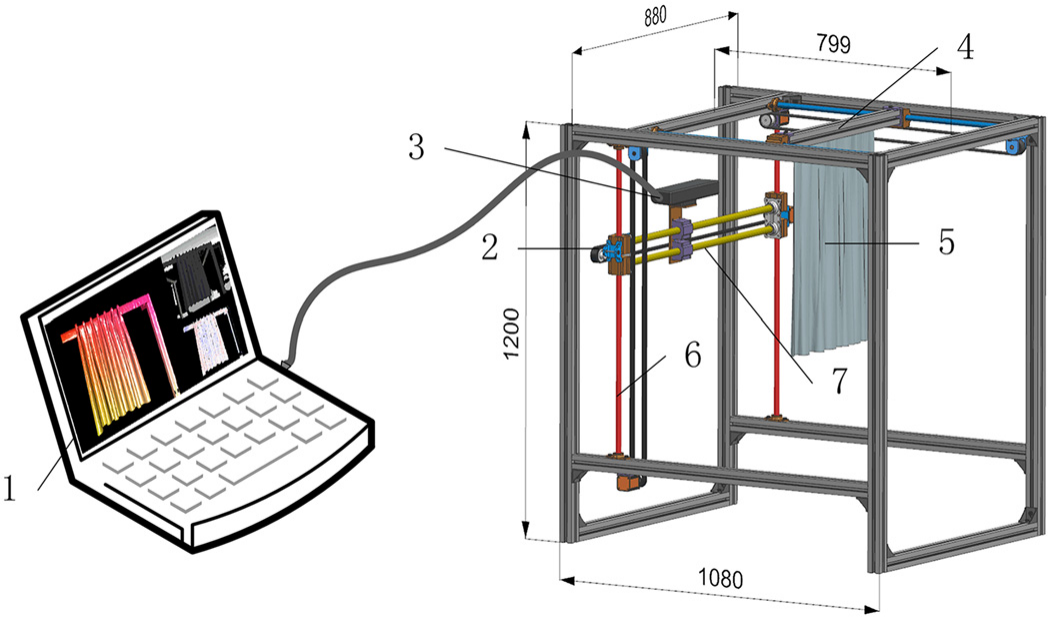
Experimental drape tester. 1-computer, 2-step motor, 3-Kinect, 4-clamp, 5-sample, 6 and 7-slide guide (all dimensions are in millimeters).

**Fig 2:**
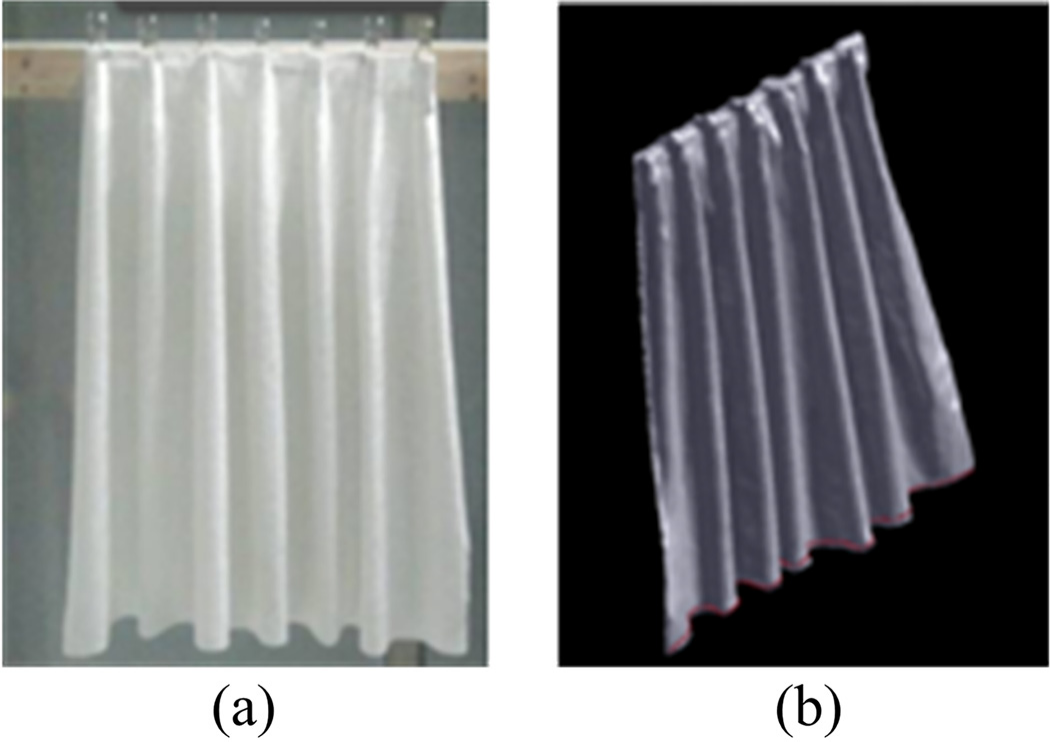
(a) Sample photo; (b) the 3D model of the sample.

The test work was carried out by a self-designed drape meter. This is a new, computer-controlled and 3D scanning based equipment. It consists of three parts: a clamp, a Kinect Sensor and a computer. The schematic diagram of the drape meter is shown in Fig 1. The sample is clamped in a manner at one end and hangs free at the other, as shown in Fig 2(A), which is the so called unidirectional fabric drape. The Kinect Sensor was mounted on slide guide and it can be driven by step motors to move side-to-side and up-to-down. The fabric sample can move forwards and backwards to adjust scanning distance. To capture clear image, the Kinect Sensor should scan the sample from different spatial locations repeatedly. Fig 2(B) shows a typical drape profile model captured by this method. The 3D model was then saved to the computer and processed by the image-analysis software to calculate unidirectional drape parameters.

The Kinect Sensor is actually a depth camera developed by Microsoft and it can enable us to capture 3D depth images. With the help of KinectFusion [22–23], a 3D point cloud splicing technology, the clear 3D model of the sample is obtained by fusing multiple depth images together in the same coordinate system.

To check the precision and repeatability of our equipment, a regular geometrical object cuboid of 562*mm* × 391*mm* × 112*mm* was chosen as the scanning object. Table 1 recorded the test data of 10 times measurements. It can be clearly seen that the relative error is about 0.3% and the maximum deviation is less than 2*mm*. Both the stability and reproducibility are very good. As for our test work, the relative error within 1% is acceptable. So the accuracy meets our test requirements.

**Table 1:** Accuracy test of the equipment.

No.	length/mm	width/mm	height/mm
1	559	388.3	113.7
2	554.1	391.9	114
3	554.4	386.6	110.5
4	568.5	391.1	111.3
5	564	392.8	113
6	562.8	387.4	112.2
7	558.6	390	110.9
8	560.2	389.9	113
9	555.6	392.4	113.1
10	567.2	391.2	111.2
Average value	560.44	390.4	112.29
Actual value	562	391	112
Absolute error	1.56	0.6	0.29
Relative error	0.28%	0.15%	0.26%
Standard deviation	5.105	2.15	1.242
Relative standard deviation	0.91%	0.55%	1.11%

### Materials

For this study 20 pure polyester woven fabric samples with different weave structures were used. All the samples were conditioned at 65±2% relative humidity and 20±2°C temperature for 24 hours before measuring to relieve localized stresses caused by handling during preparation. We selected these samples because they are common in clothing fabrics and commercially available and less affected by temperature and humidity. The detailed physical properties of the samples are listed in Table 2.

**Table 2:** Basic properties of sample fabrics.

Fabric	Weave structure	Thickness (mm)	Weight(g/m^2^)	Density (picks/inch)		Bending stiffness(cN.cm)	
				Warp	Weft	Warp	Weft
1	Plain	0.8	215	96	84	0.078	0.088
2	Plain	4.542	321	120	100	0.23	0.226
3	Plain	0.722	65	105	77	0.006	0.014
4	Plain	2.264	232	53	65	0.071	0.045
5	Plain	0.324	78	121	99	0.058	0.019
6	Plain	0.516	195	96	80	0.005	0.007
7	Plain	0.496	166	119	102	0.178	0.065
8	Plain	0.73	120	99	84	0.098	0.034
9	1/2 twill	1.238	167	112	85	0.119	0.037
10	1/2 twill	0.98	168	118	78	0.268	0.037
11	1/2 twill	1.5	262	101	101	0.216	0.109
12	1/2 twill	0.68	53	118	76	0.051	0.081
13	1/4 twill	0.506	133	111	95	0.066	0.067
14	1/4 twill	0.962	72	96	73	0.014	0.013
15	1/4 twill	0.704	81	66	80	0.046	0.027
16	Satin	1.424	268	122	84	0.079	0.076
17	Satin	0.658	104	124	102	0.089	0.038
18	Satin	0.16	71	147	158	0.017	0.018
19	Satin	0.472	103	113	91	0.1	0.051
20	Satin	0.134	78	89	105	0.012	0.041

## Method

**Fig 3:**
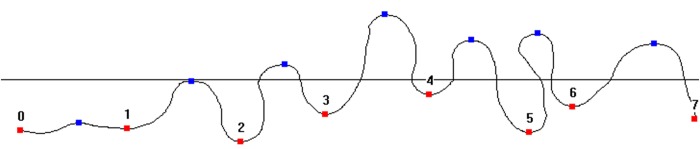
Projective wave curve.

In this study, we chosed the projective wave curve (as the red curve shown in Fig 2(B)) which was obtained by intercepting the 3D model with a horizontal plane from the bottom of the fabric as our evaluating object for the following reason: the closer the horizontal plane was to the holder, the stronger clamping effect to the sample would be, which meant that the fabric drape was not in a free hanging state and the result would not be accurate. Fig 3 is the projective wave curve we extracted. Through recognizing the peaks and the troughs (the blue and red points in Fig 3) of the wave curve, we can calculate unidirectional drape parameters such as unidirectional drape coefficient (*UDC*), wave numbers, bending index, peak height and peak width. Here the *UDC* is a new parameter defined by Eq (1). It is created for the description of drape degree, which indicates that the smaller the value of *UDC*, the better the drape of the fabric.

$$UDC=\frac{L_{0}-L_{2}}{L_{0}-L_{1}}\times 100\%$$ (1)

**Fig 4:**
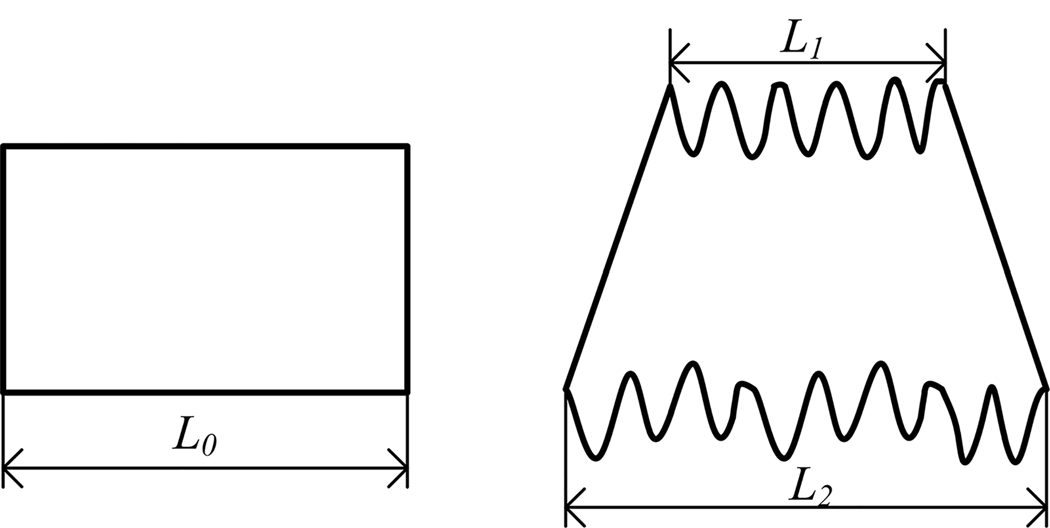
The definition of *UDC*.

Where *L*_0_ is the length of the specimen, *L*_1_ is the folding width and *L*_2_ is the draping width, as shown in Fig 4.

The bending index refers to the ratio of the curve length between two adjacent troughs (such as point 2 and point 3 in Fig 4) and their horizontal distance, which is used for describing the degree of the drapability. The wave numbers, peak height and peak width are also drape degree parameters and their definitions have no difference with the conventional drape parameters.

### Fabric Height

**Fig 5:**
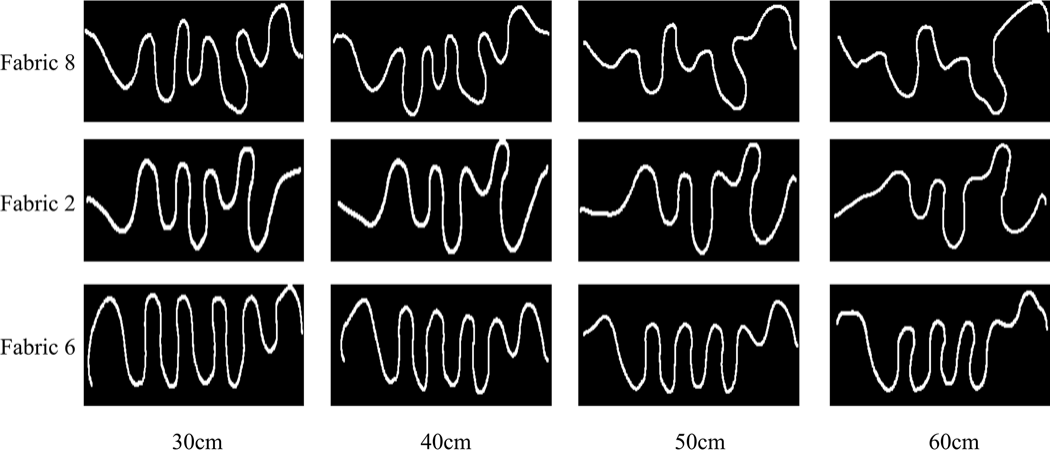
Different projective wave curves at different fabric height.

It is obvious that the shape of fabric will be distinctly different when the fabric height changes. As the fabric height decreases, the clamping effect will be increasingly strong. But on the other hand, fabric shapes will be very random and the bottom crinkle tends to be a straight line if the height is too large. In order to choose the most proper height value, we made a comparison among projective wave curves of the 20 fabrics at the height of 30cm, 40cm, 50cm and 60cm and selected 3 fabrics (see Fig 5) whose drape shape changed the most significantly.

As shown in Fig 5, with the decrease of fabric height, the projective wave curves tend to be similar. The wavenumbers are the fewest at the height of 60cm and the uniformity is the worst. The curve shapes of three fabrics change not very obviously at the height of 30cm and 40cm, which makes it difficult to discriminate good from bad drape performance among different samples. And when the height is set as 50cm, it is clear that the distinctions among three fabrics are the most obvious. Through above analysis, the sample height was selected 50cm finally. As for the fabric width, it correlates with unidirectional drape not very closely and in this study we set it as 87cm considering our equipment size.

### Clamping Manner

**Fig 6:**
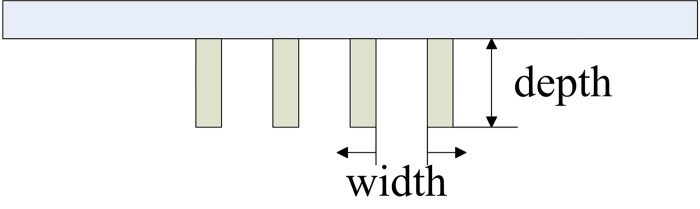
Clamping depth and width (top-view).

The clamping manner involves two aspects including clamping depth and width (Fig 6) and it has a significant effect on the unidirectional drape performance. It is difficult to form wave curve if the clamping depth is too small because of short wave amplitude, but on the contrary, too large clamping depth will reduce wavenumbers and form more regular wave curve, which means that the differences of curve shapes among fabrics will be less obvious. With the increase of clamping width, the wavenumbers will be fewer and fewer; the wave curve will overlap if the clamping width becomes too narrow, which makes it difficult for Kinect to capture the complete fabric contour.

**Fig 7:**
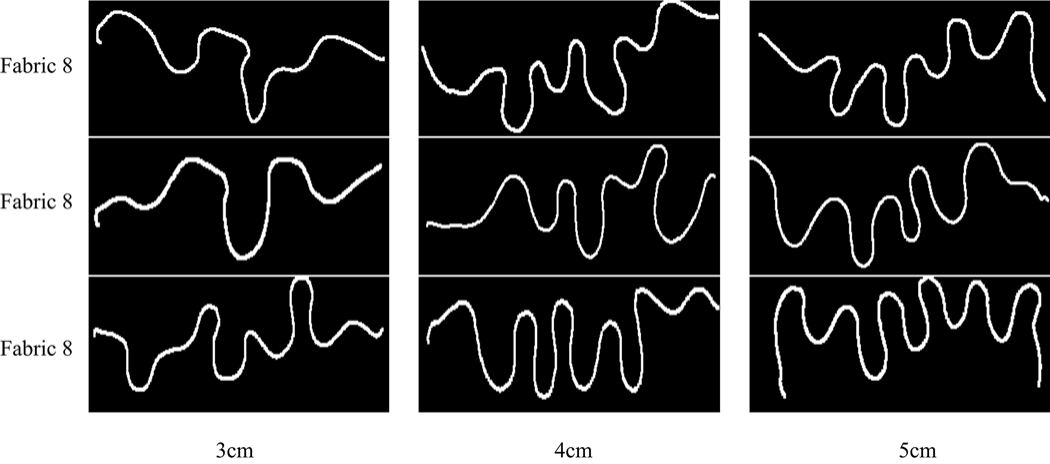
Different unidirectional drape projection wave curves at different clamping depth.

**Fig 8:**
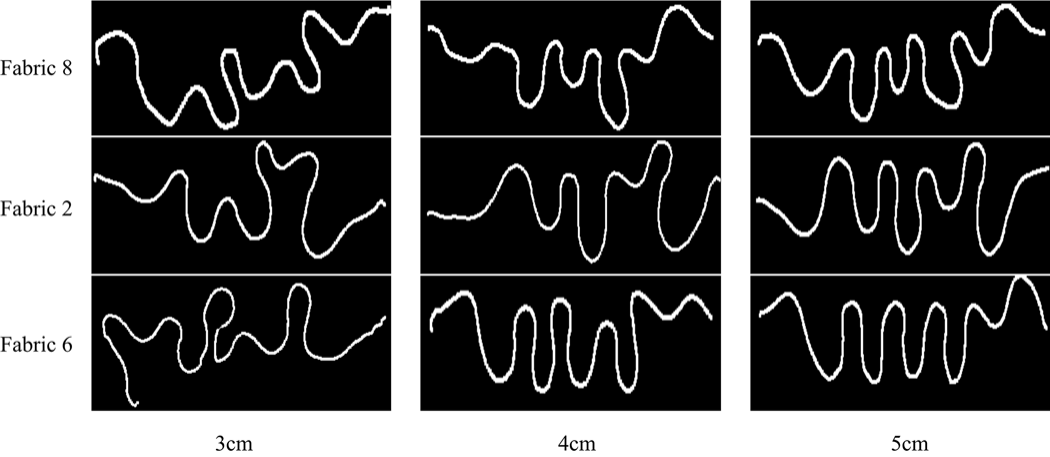
Different unidirectional drape projection wave curves at different clamping width.

In order to select an appropriate value of clamping depth and width, we set the clamping depth and width as 3cm, 4cm and 5cm respectively considering the fabric width. We captured the projective wave curve of three fabrics mentioned above at the clamping depth and width of 3cm, 4cm, and 5cm respectively and the results are listed in Figs 7 and 8. We can see that larger clamping depth and width value give more regular shapes and smaller differences among fabrics. The wavenumbers are the fewest and the wave shapes are not obvious especially in *Fabric* 8 and 2 at the clamping depth of 3cm and the clamping width of 3cm. As for clamping depth and width of 4cm and 5cm, the latter is more difficult to compare unidirectional drape differences among three fabrics than the former. So clamping depth and width of 4cm is better recommended value for the unidirectional draping measurements.

## Results and Analysis

This study employed CNS (Chinese National Standard) *GB/T23329-2009 Textiles*. *Determination of drapability of fabrics* to represent the conventional method and performed comparisons with the novel method proposed in this paper. Tables 3 and 4 showed the drape parameters of 20 fabrics of both methods. We only listed the main drape degree and aesthetics parameters that determine drape performance to a large extent according to previous researches.

**Table 3:** Experimental results of fabrics drape by conventional method.

Fabric	Drape coefficient (%)	Angle irregularity (%)	Peak height irregularity	Peak width irregularity (%)
1	47.3	9.255	10.471	9.726
2	90.8	8.977	11.096	11.404
3	26.8	10.051	7.507	6.695
4	58.3	7.051	6.048	4.245
5	48.9	10.794	4.942	7.428
6	12.4	2.187	7.985	7.3
7	79.6	5.347	7.327	8.684
8	76.2	11.173	1.789	3.427
9	32.2	5.753	7.229	3.566
10	85.6	6.618	3.388	3.812
11	77.2	6.168	10.116	10.518
12	63.5	5.377	6.301	8.007
13	72.6	3.816	6.341	6.579
14	64.4	10.08	4.92	4.44
15	71.4	7.667	6.4	1.693
16	54	9.953	1.481	1.2
17	78.6	11.77	6.716	5.751
18	69.4	9.514	1.582	2.105
19	74.4	6.618	4.134	7.659
20	42.3	6.632	2.094	5.171

Here, the angle irregularity refers to the variation coefficient of the angle between two adjacent peaks and it is calculated as the following formula.

$$\hat{\theta }=\frac{1}{\bar{\theta }}\sqrt{\frac{1}{n-1}\sum_{i=1}^{n}{\left(\theta_{i}-\bar{\theta }\right)}^{2}}\times 100\%$$ (2)

**Fig 9:**
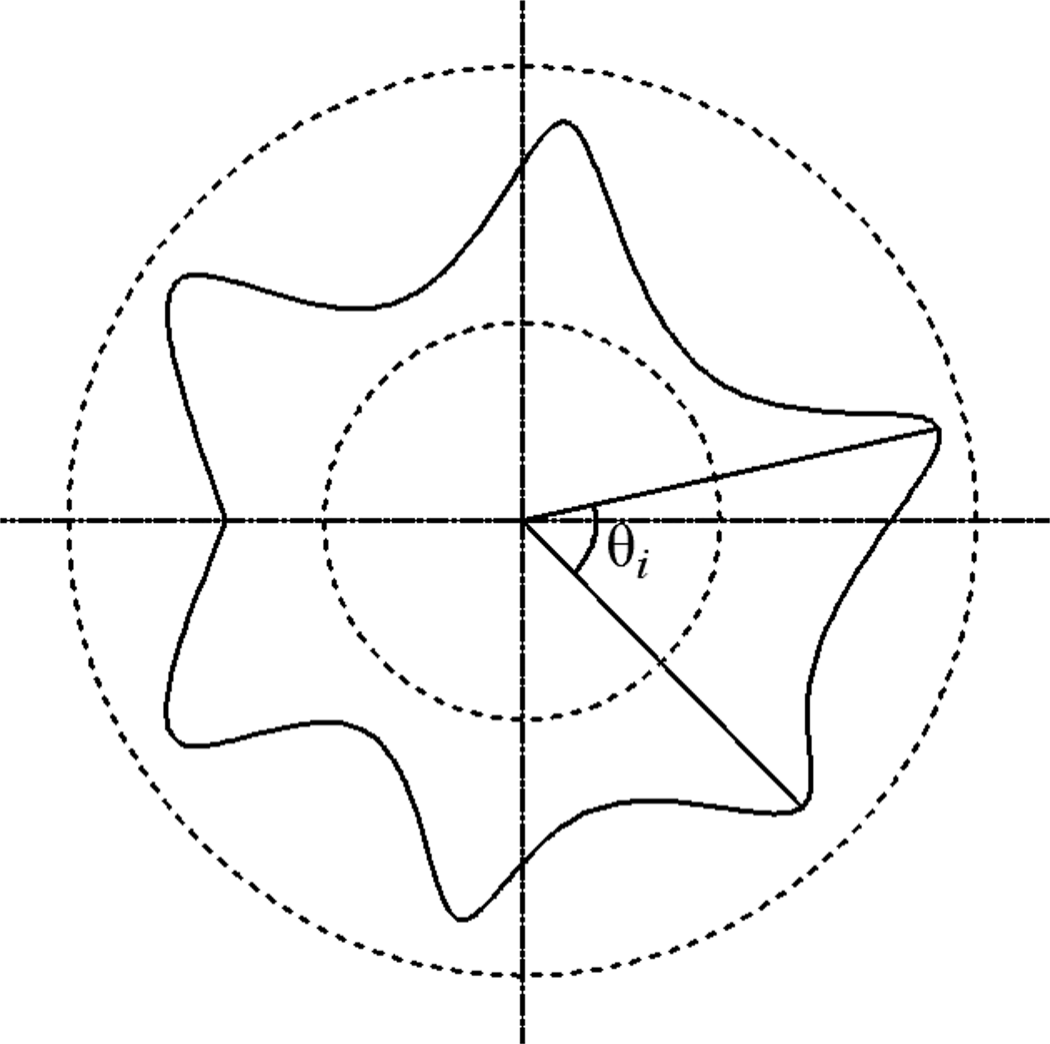
Draped fabric specimen.

Where $\hat{\theta }$ is the angle irregularity, $\bar{\theta }$ is the average value of peak angle, *θ*_*i*_ is the angle value between neighboring fold peaks (see Fig 9).

**Table 4:** Experimental results of fabrics drape by unidirectional method.

Fabric	*UDC* (%)	Peak numbers	Avg. bending index	Bending variation index (%)	Avg. peak height(mm)	Peak height variation index (%)	Avg. peak width (mm)	Peak width variation index (%)
1	76.45	6	2.077	7.39	40.126	8.073	40.256	5.922
2	44.84	4	7.901	17.861	63.326	12.996	57.027	6.62
3	89.7	6	2.577	3.823	45.716	14.627	49.082	8.125
4	75.6	6	2.684	6.419	50.709	6.002	46.061	6.265
5	80.27	7	2.594	5.474	44.889	4.155	52.939	5.651
6	92.78	7	2.387	2.686	40.115	6.657	55.764	7.513
7	48.87	5	1.811	12.179	43.628	11.083	43.096	12.532
8	59.31	6	2.124	12.95	41.789	10.013	57.331	8.652
9	87.83	7	2.193	4.622	42.692	7.015	49.032	7.201
10	56.52	5	2.222	11.608	47.591	10.564	48.64	9.562
11	54.3	5	2.148	8.001	50.058	11.688	49.18	9.628
12	69.57	5	2.263	13.022	47.391	7.877	51.315	11.526
13	60.87	5	2.385	12.722	45.051	9.233	60.281	7.654
14	72.91	7	2.263	8.975	39.924	4.444	47.908	9.854
15	66.45	7	2.265	10.816	41.352	7.85	52.5	7.485
16	77.36	7	2.743	11.884	50.413	5.077	46.261	5.598
17	57.68	6	2.317	10.651	42.783	10.498	57.755	8.486
18	64.77	6	2.397	10.822	45.359	6.139	43.143	3.634
19	63.26	6	2.656	10.179	45.924	8.187	54.166	7.233
20	84.52	7	2.748	7.881	42.506	5.211	51.351	10.621

We cannot compare these two methods straightly since the testing principles of these two methods are completely different. So a comprehensive index describing fabric drape is needed. In this research, we calculated the weighting coefficients of each drape parameters with the main-factor analysis of SPSS 17 software and a comprehensive index, namely aesthetic coefficient, was obtained and given as Eqs (3) and (4), respectively.

$$B_{1}=0.461f+0.109\hat{\theta }+0.14\hat{h}+0.207\hat{d}$$ (3)

Where *B*_1_ is the aesthetic coefficient of conventional method, *f* is the drape coefficient, $\hat{\theta }$ is the angle irregularity, $\hat{h}$ is peak height irregularity, and $\hat{d}$ is peak width irregularity;

$$B_{2}=0.553f_{u}+0.243\theta +0.168h$$ (4)

Where *B*_2_ is the aesthetic coefficient of unidirectional method, *f*_*u*_ is the unidirectional drape coefficient, *θ* is the bending variation index, and *h* is the peak height variation index.

**Fig 10:**
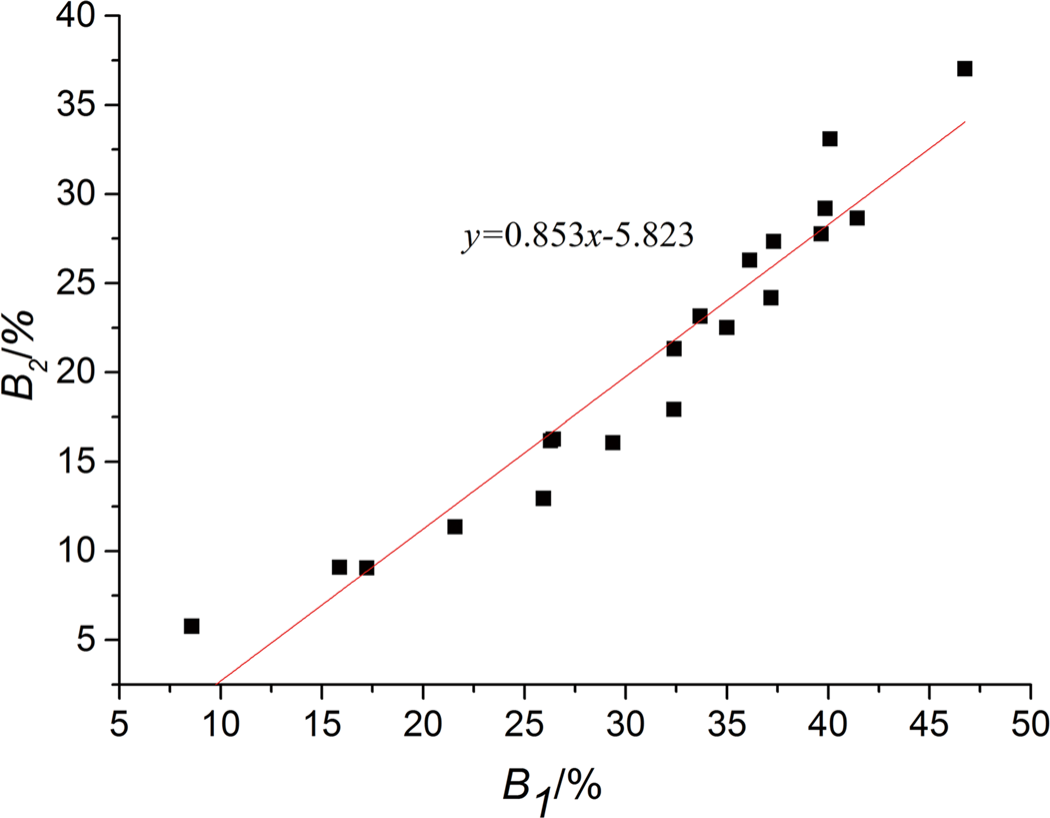
Relationships between conventional method and unidirectional method. *B*_1_ is the aesthetic coefficient of conventional method; *B*_2_ is the aesthetic coefficient of unidirectional method.

Linear regression analysis was used to evaluate correlations between *B*_1_ and *B*_2_ and the result was shown in Fig 10. The correlation coefficient at the 1% level of significance was 0.964 which showed a highly correlated linear relationship between *B*_1_ and *B*_2_, in another words, the unidirectional method is quite consistent with conventional method in drape parameters given by these two methods. So the unidirectional method is acceptable and equivalent to the conventional method in describing fabric drape performance.

**Fig 11:**
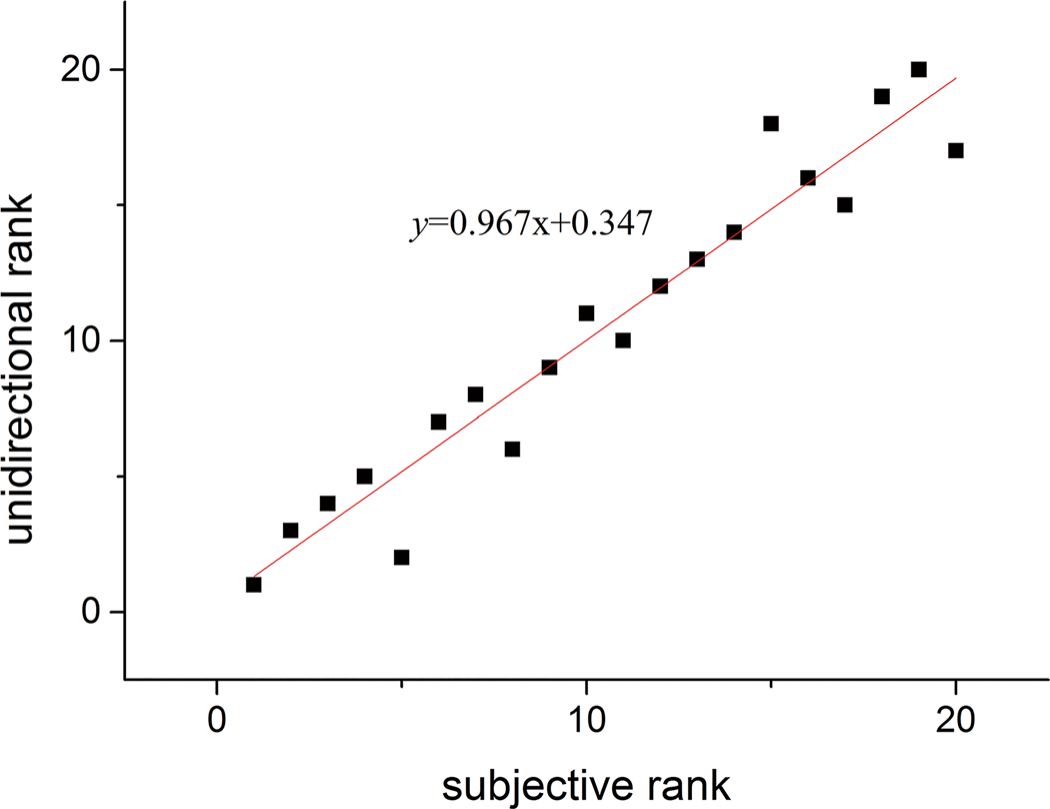
Relationships between unidirectional method and subjective evaluation.

Drape is a subjective property and results produced by drape meters should correlate with subjective evaluation [14]. In this research, we invited 10 professionals and 10 non-professionals to evaluate drape performance. The drape performance was divided into 5 grades from bad to good. The scores range from 1 to 20 where 20 represents the best of drape performance and 1 is the worst. The evaluators graded each samples through judging the drape degree and aesthetic feeling of drape shapes using their visual sense. The final score is calculated as this formulation: (professionals×60%+non-professionals×40%)/10. We sorted the final score and *B*_2_ calculated above and listed the ranking results in Table 5. In order to determine the relationships between subjective evaluation and unidirectional method, we made a linear regression analysis of score rank and *B*_2_ rank as shown in Fig 11. The correlation coefficient at the 1% level of significance was 0.967 and it can be clearly seen that unidirectional method correlates strongly with subjective method in evaluation of fabric drape. So there is no sig0nificant difference between these two methods in describing fabric drape.

**Table 5:** Subjective evaluation and ranking results.

Fabric	Sum scores of 10 professionals	Sum scores of 10 non-professionals	Final score	Score rank	*B*_2_ rank
1	151	161	15.5	6	7
2	19	21	1.98	19	20
3	181	176	17.9	2	3
4	135	131	13.34	8	6
5	168	167	16.76	4	5
6	200	197	19.84	1	1
7	25	23	2.42	18	19
8	46	44	4.52	17	15
9	162	157	16	5	2
10	17	17	1.7	20	17
11	55	56	5.54	15	18
12	95	89	9.26	11	10
13	80	83	8.12	14	14
14	121	118	11.98	9	9
15	105	115	10.9	10	11
16	138	139	13.84	7	8
17	48	49	4.84	16	16
18	92	87	9	12	12
19	84	93	8.76	13	13
20	178	177	17.76	3	4

## Conclusion

A new fabric drape testing method was presented and corresponding drape meter with automatic measuring system was devised. This is a new, low-cost, non-contact scanning and 3D measurement method.

A new parameter, unidirectional drape coefficient (*UDC*), which is used to describe drape degree, was created in our work. And the other unidirectional parameters such as peak height, peak width, bending index and wavenumbers were obtained from the 3D fabric contour.

In order to investigate the feasibility of the unidirectional testing method, we compared the result of unidirectional method with those of conventional method and subjective evaluation method respectively. It confirmed that the unidirectional method was quite consistent with conventional method and subjective evaluation. Thus unidirectional fabric drape testing method can be implemented on a rapid and automatic basis to enable full characterization of the drape profile of fabrics.

Unidirectional fabric drape testing method has some advantages over conventional method. First, this method simulates the mechanical conditions of fabric drape in actual situation where the gravity is parallel not vertical to fabric plane, so the results of unidirectional drape testing method is closer to that of real. Second, this is a 3D measurement and it can reflect drape performance more completely. Third, the fabric drape is not influenced by fabric transmittance. But this method only uses polyester material and woven fabrics as our testing materials. We will test unidirectional drape of different materials and fabric structures to further confirm the feasibility of this new method. And we do not discuss which factors and how they influence unidirectional drape performance because of limited time. The subsequent study will put emphasis up on discussing factors influencing unidirectional drape to find relationships between these factors and unidirectional drape.
